# Research progress on the development of pennycress (*Thlaspi arvense* L.) as a new seed oil crop: a review

**DOI:** 10.3389/fpls.2023.1268085

**Published:** 2023-11-29

**Authors:** Jianyu Ma, Haoyu Wang, Yuhong Zhang

**Affiliations:** ^1^ Key Laboratory of Forest Plant Ecology, Ministry of Education, Northeast Forestry University, Harbin, China; ^2^ Heilongjiang Provincial Key Laboratory of Ecological Utilization of Forestry-Based Active Substances, Harbin, China

**Keywords:** *Thlaspi arvense* L., supplementary model plant, gene, seed oil, fatty acid, germplasm development, abiotic stress

## Abstract

Compared with other crops, pennycress (*Thlaspi arvense* L.) is a niche emerging oil crop. In recent years, research on pennycress has been increasingly reflected in various directions. Pennycress belongs to the *Brassicaceae* family and was introduced from Eurasia to North America. It has been found worldwide as a cultivated plant and weed. In this paper, we review the advantages of pennycress as a supplementary model plant of *Arabidopsis thaliana*, oil and protein extraction technology, seed composition analysis based on metabolomics, germplasm resource development, growth, and ecological impact research, abiotic stress, fatty acid extraction optimization strategy, and other aspects of studies over recent years. The main research directions proposed for the future are as follows: (1) assemble the genome of pennycress to complete its entire genome data, (2) optimize the extraction process of pennycress as biodiesel, (3) analyze the molecular mechanism of the fatty acid synthesis pathway in pennycress, and (4) the functions of key genes corresponding to various adversity conditions of pennycress.

## 1 Introduction

Energy crisis has become a global problem to be solved urgently. The promotion of industrial development and the exploitation of oil have led to the near depletion of oil resources. The ever-growing energy consumption and the associated environmental issues have urged for new sources of energy. With the development of science, technology, and industry, oil resources are increasingly limited. Finding alternative and environment-friendly oil resources to meet the needs of social development has become an urgent problem. Biodiesel is a kind of an environment-protective energy source. *Thlaspi arvense* L., as a new oil crop, has attracted much attention. *T. arvense* L., with alternative names of pennycress, field pennycress, stinkweed, bastard cress, field thlaspi, and ail sauvage, belongs to *Brassicaceae* and introduced to North America from Eurasia (Warwick et al., 2002). This cultivated plant species is also found as a weed in other places of the world. Pennycress can be grown in different regions of the world to produce food, feed, and fuel (including renewable aviation fuel) and benefit the ecosystem by providing sustainable living coverage (Marks et al., 2021; Mousavi-Avval and Shah, 2021b; Keadle et al., 2023). An abundance of scientific evidence shows that mankind must achieve carbon neutrality by 2050 or even earlier to protect civilization and ecosystems from the devastating consequences of climate change. To achieve this, we must not only reduce and replace the use of fossil fuels, such as biofuels, but also need carbon capture and storage (CCS) to restore the carbon dioxide in the atmosphere to the current level.

**Figure 1:**
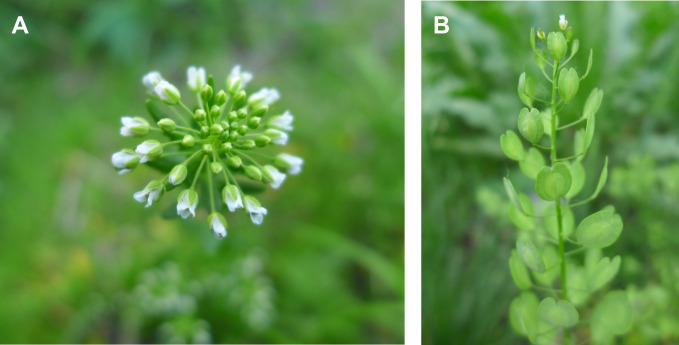
Pennycress growth morphology in the wild. **(A)** Raceme, petals white, spatulate, apex obtuse or emarginate. **(B)** Fruit obovate or suborbicular, base obtuse or rounded, apex deeply emarginate and apical notch.

Many genes replicated in *Arabidopsis* exist in the form of a single copy in mustard, which indicates that it will be easier to study their functions in mustard (McCormick, 2018). *T. arvense* is relatively easy to grow with a larger biomass than *Arabidopsis*, so it is more suitable for biochemistry development and other research (**Figure 1**). *T. arvense* can achieve 93.4% carbon conversion efficiency (CCE), which is much higher than other oilseeds (Sedbrook and Durrett, 2020). It has been estimated that once planted in half of the US Midwest Corn Belt, pennycress could fix 40 Mt of carbon and yield 9.8 billion liters of oil and 17 Mkg of seed meal each year (Fan et al., 2013).

At present, there is still a lack of in-depth research on pennycress. On the basis of existing research in recent years, this paper briefly reviewed the research on pennycress in the aspects of chromosome level detection, ecological development and distribution, abiotic stress, germplasm resource development, fatty acid composition, etc., to assist in the research on pennycress.

## 2 Pennycress is expected to become a supplementary model plant of *Arabidopsis*

Although *Arabidopsis thaliana* has been widely used as a model plant, it is not eligible as a model plant to study crop performance problems due to its short stature and lack of agronomic value. Over the past decade, numerous studies have demonstrated that pennycress can serve as an alternative model system analogous to *Arabidopsis* as it is desirable for both laboratory tests and large-scale experiments in the field. In recent years, a method similar to *Arabidopsis thaliana* for the genetic transformation of pennycress through *Agrobacterium*-mediated vacuum infiltration has been developed, which can produce 0.5% transformed seeds (McGinn et al., 2019). The *TaFAE1*‐CRISPR‐Cas9_Hyg vector was introduced into pennycress plants using the method described above to produce mutations in the *FATTY ACID ELONGATION1* (*FAE1*) gene, therefore producing an edible seed oil. After having been transformed with the gene that controls *Euonymus alatus* diacylglycerol acetyltransferase (*EaDAcT*) using the soybean glycinin promoter, the crop would be able to produce a novel drop-in fuel for diesel engines with low viscosity.

Numerous approaches are used to dissent the molecular networks of *T. arvense*. A research has sequenced, assembled, and annotated the transcriptome of pennycress (Dorn et al., 2013). Of these transcripts, 35% were most similar to an *A. thaliana* gene, and 74% were with top hits to the *Brassicaceae*. To generate a draft genome of *Thlaspi arvense* line MN106, a hybrid sequencing approach was used to generate 47 Gb of DNA sequencing reads from both the Illumina and PacBio platforms, which was annotated using the MAKER pipeline (Dorn et al., 2015). Compared with other *Brassicaceae* species, pennycress gene homologues revealed a high sequence global conservation that especially participated in glucosinolate biosynthesis, metabolism, and transport pathways. It was found that the peptides of *T. arvense* and five cruciferous plants including *Arabidopsis thaliana*, *Arabidopsis lyrata*, *Brassica rapa*, *Capsella rubella*, and *Eutrema salsugineum* were highly conserved through a combination comparative analysis, and *E. salsugineum* possessed the highest proportion of highly similar predicted peptides. EMS is used as a mutagen to induce *T. arvense* mutation, resulting in a pool of thousands of mutation genes, most of which are traceable in *Arabidopsis* (Chopra et al., 2018). The reduction of gene redundancy has promoted the identification of characters in *T. arvense* and the production of *T. arvense* mutants highly similar to *Arabidopsis* (Sedbrook et al., 2014).

A recent study suggested that the genome assembly of *T. arvense* var. MN106-Ref at the chromosome level with improved gene annotation can produce abundant variants, which represent both the genetic diversity in the collection and species population structure (Nunn et al., 2022). A sufficient variation in the transcripts was observed from two separate lines—MN108 and Spring32-10—during the analysis of transcriptome sequences. To avoid spurious associations and false positives, a unified mixed-model method is adopted, taking into account the population structure and kinship (Tandukar et al., 2022). It is the first report that specifically focuses on comprehending the genetic control of secondary domestication traits like seed size, oil content, and protein in pennycress populations in a multi-environment study. The results confirmed that genome-wide association study (GWAS) was an effective strategy to identify significant marker trait association, which was helpful for the breeding of pennycress.

## 3 Evaluation of the extraction technology of pennycress seed oil and protein

The pennycress seed contains 36% oil, which has erucic acid as its largest fatty acid (33%–38%), with linoleic and linolenic acids as the other two major components. (Moser et al., 2009b; Hojilla-Evangelista et al., 2013). It was found that pennycress oil could be converted into field pennycress oil methyl esters (FPME) (biodiesel) by a traditional alkali-catalyzed technique (Moser, 2012). In the assessment of the life cycle of pennycress jet fuel and diesel, a scenario analysis of the nitrogen fertilizer application amount, nitrogen content in crop residua, sources of H_2_, and direct land use change (dLUC) has proved that the biofuels derived from *T. arvense* met the Renewable Fuel Standard (RFS2) and are promising to be widely used as advanced biofuels and biomass-based diesel (Fan et al., 2013; Moser et al., 2016; Mousavi-Avval and Shah, 2021a).

Compared with biodiesel extracted from commercial lipids, for instance, soybean, camelina, canola, sunflower, especially palm oils (Moser et al., 2016), the biodiesel produced has many splendid features, such as low cold filter plugging point (−17°C), cloud point temperature (−10°C), and pour point (−18°C) (Moser, 2012). Thereinto, the most important is the high cetane number and outstanding cold flow property (Moser et al., 2009b). Moser et al. (2009a) evaluated pennycress oil for the first time and evaluated that it conforms to the regulations of biodiesel according to the United States American Society for Testing and Materials (ASTM D6751). Nevertheless, the presence of longer-chain fatty acid methyl esters (FAME) has led to the kinematic viscosity of FPME being 5.24 mm^2^/s at 40°C, which exceeds the 3.5–5.0 range specified in the European Committee for Standardization (CEN, EN 14214) (Moser, 2009; Moser et al., 2009a; Dunn, 2022). Therefore, for the purpose of better ameliorating the biodiesel characteristics of pennycress, it can be mixed with diesel oil with low kinematic viscosity to meet the EN 14214 (Moser et al., 2009a; Moser et al., 2016).

Isbell et al. (2015b) first concentrated erucic acid and methyl erucic acid by molecular distillation. The residence time of the molecular distillation unit is short under high temperatures, so it can distill macromolecular compound fatty acids (such as fatty acids or the corresponding methyl esters) without heat degradation like other distillation units, with rapid reaction and low cost (Isbell et al., 2015b).

The amino acid (AA) component of the protein in *T. arvense* is a typical plant protein, in which glycine, glutamic acid, and alanine are ubiquitous, with low-level essential amino acid content (Selling et al., 2013). The protein content produced by using 0.5 M sodium chloride at 5°C is the highest, reaching 83%, which is categorized to concentrate, while the extraction rate was only 25%. Pennycress crude protein extract had medium solubility (35%–45% soluble protein at pH 4), but it shows other excellent characteristics— foaming ability and foam stability (93%–97% residual foam) equalized with soybean protein and emulsification ability superior to lesquerella seed and the press cake (EAI > 150 m^2^/g protein) (Hojilla-Evangelista et al., 2013; Hojilla-Evangelista et al., 2014). Thus, it is necessary to improve the extraction process to obtain a higher protein yield.

Two methods of protein extraction from pennycress (*T. arvense*) seed meal were evaluated, and the constituent, amino acid distribution, and chemical and physical properties (e.g., solvability, foamability, emulsifying, water holding capacity, and thermal coagulation) were compared with those of the synthesized protein (Hojilla-Evangelista et al., 2014; 2015). The results showed that the extraction method had an important effect on the purity and chemical and physical properties of the pressed protein products. Salt alkali precipitation (SE) was carried out through 0.1 mol/L NaCl at 50°C, while the traditional alkali dissolution and acid precipitation (AP) related to alkaline extraction (pH 10) were first followed by protein precipitation at pH 4. The crude protein extracted by SE and AP was at least 90% (db), which was the protein isolates (PI). A comparison of the results reveals that APPI had a lower protein yield (23%) but with a much higher purity (90% crude protein) than SE (45% yield and 67% crude protein) (Hojilla-Evangelista et al., 2014). Meanwhile, APPI showed higher foam capacity (120 ml), foam stability (96% foam volume retention), emulsification stability (24–35 min), and excellent heat resistance (solubility loss of 3% at pH 2 and pH 10). SEPI has better solubility (68%–91% at pH 2 and ≥7) and has a significantly competitive emulsifying activity than APPI (226–412 m^2^/g protein). In short, SE and AP can extract protein isolates with outstanding characteristics from the seeds of *T. arvense*. Evangelista et al. (2012) determined that seed moisture content (MC) influences the pressing quality parameters of *T. arvense* seeds. Full-pressing and cooking have negative effects on the phosphatides and sulfur content in grains but have no effects on free fatty acid levels and oil color. The whole pennycress seed containing about 10% MC can be squeezed with minimum seed preparation conditions to obtain a press cake successfully, with an oil extraction rate of 10.7% (db) and 75.1% of the seed oil. Cooking and drying the seed MC between 3% and 4% offered a maximum oil recovery rate of 86.3% and 88.0%, respectively. The fast pyrolysis of defatted seed meal created stable high-carbon, low-oxygen (<30 wt%), high-energy liquid fuel intermediates that can manufacture jet fuels (Boateng et al., 2010; Evangelista et al., 2012). Transportation is one of the main cost reduction segments in the supply chain of producing sustainable aviation fuel (SAF) using pennycress (Trejo-Pech et al., 2019). A research has developed a GIS-based model that can provide suitable refinery site selection for SAF biorefineries based on pennycress (Mousavi-Avval et al., 2023). Although the final use of pennycress oil is mainly embodied in biodiesel, it is also planned to target the non-fuel industrial chemical marketplace and edible oil marketplace (Isbell et al., 2015a; Altendorf et al., 2019). Zhao et al. (2021) used the microwave-assisted biphasic extraction one-pot method for the first time to effectively extract oil and sinigrin from the seeds of pennycress.

## 4 Analysis of pennycress seed components based on metabolomics

The natural accumulation of erucic acid in *T. arvense* renders it as an outstanding oil crop and industrial crop. Metabolite fingerprints of the physiological activity of *T. arvense* embryos were analyzed by using non-targeted metabolomics and elective quantification of pivotal intracellular metabolites (more targeted) (Tsogtbaatar et al., 2015). A gas chromatography–mass spectrometry (GC–MS) analysis showed that there were three compound families of intracellular metabolites: organic acids (mainly malic acid and citric acid), amino acids, and sugar/sugar alcohols. Liquid chromatography tandem mass spectrometry (LC–MS/MS) was used to analyze the compounds obtained from boiling water extraction of different developmental stages of pennycress embryos. The results explored that glycolysis, oxidative pentose phosphate pathway, tricarboxylic acid cycle (TCA), and Calvin cycle were active in the growth process of pennycress.

According to the composition of liquid endosperm, Tsogtbaatar et al. (2020) established an *in vivo* culture system similar to the embryonic growth of apetala in plants. The biosynthetic efficiency of cultured pennycress embryos was measured as 93%, which was one of the highest compared with other oilseeds so far. The parallel labeling experiment with ^13^C-labeled substrate in pennycress *T. arvense* revealed four reactions involved in fatty acid synthesis and extension. One is the oxidation reaction of the pentose phosphate pathway in the cytoplasm. The other is that isocitrate dehydrogenase (IDH), traditionally regarded as a catalytic and thermodynamic irreversible decarboxylation reaction, is reversible in pennycress embryos. Third, NADP-dependent malic enzyme (NADP-ME) generates pyruvate through the decarboxylation of malic acid transported in plastids. Fourth, Rubisco recycles carbon dioxide released by plastic pyruvate dehydrogenase (PHD) and malic enzyme, meanwhile providing a carbon skeleton for *de novo* petroleum synthesis. It should be noted that environmental impact should also be considered when evaluating breeding materials and predicting overall performance.

## 5 Development of germplasm resources of pennycress

Nowadays, the agricultural system in the Midwest of the USA is mainly a corn and soybean double-crop system (Johnson et al., 2017). The introduction of new species in this traditional agricultural system can bring higher economic and ecological benefits. During the fallow period, the potential production of *T. arvense* seeds is 1,120–2,240 kg · ha^–1^, equivalent to 600–1,200 L · ha^–1^ of oil, while the potential yield of soybean and camellia seed oil is 450 and 420–640 L · ha^–1^, respectively (Boateng et al., 2010; Phippen and Phippen, 2012). According to the 2-year (2009 to 2010) study by Phippen and Phippen (2012), the residue of pennycress in the field has no significant negative impact on the fatty acid content and other biomass of follow-up soybean crops. In terms of winter–spring production of fuel, *T. arvense* has considerable prospects. The experiments in the laboratory and the experimental plots proved that when 1.0 wt% of pennycress seed meal was integrated into the soil, it could effectively inhibit the germination and growth of weed seeds and would not replace the production of food crop and soybean (Isbell, 2009). Consequently, *T. arvense* can rotate with commercial crops without replacing them, and no extra land is required. Pennycress is known to exhibit both spring and winter types, sometimes within the same accession (Altendorf et al., 2019). Isbell et al. (2015a) reported that the seed germination rate of the first public nondormant pennycress winter line Katelyn (reg. no. GP-35, PI 673443) pennycress was 91% at immediate post-harvest, which was much higher than that of its original population Beecher (PI 672505) (only 7%). In spite of the fact that the germination rate of freshly harvested Katelyn (81%) seeds under light is much higher than that of the parent population (0%), it still does not meet the requirements of agricultural production (Isbell et al., 2017). Hence, an additional germplasm, Elizabeth (reg. no. GP-36, PI 677360), was developed (Isbell et al., 2017). According to the test, the germination rate of Elizabethan seeds in continuous darkness can reach 98% (Isbell et al., 2017). Elizabethan retains a winter type, the seeds need to be sowed after vernalization, and various mechanized methods are required for planting, such as unmanned aerial vehicles. Moreover, conventional herbicides can easily inhibit sprouted pennycress seeds (Isbell et al., 2015a). In another investigation, 41 winter type pennycress were collected from the United States Department of Agriculture (USDA) National Plant Germplasm System (NPGS) and wild. The yield rate of total oil, fatty acid distribution, and hundred seed weight were tested. It was found that although there were significant differences, there was no extreme variation, and mutation or additional germplasm resources were needed for domestication and improvement needed for breeding (Altendorf et al., 2019). A conserved CLAVTA3/ESR-related peptide family was identified in pennycress, including 27 gene members, most of which are involved in regulating biological processes such as root growth and shoot apical differentiation, regulating vascular bundle development, and affecting flowering and seed yield (Hagelthorn and Fletcher, 2023).

To sum up, finding novel traits and developing new breeding techniques for pennycress are necessary research directions in the future.

## 6 Research on growth and development and ecological impact

*T. arvense* belongs to the same family as *A. thaliana*, the *Brassicaceae*. Previous whole-genome sequencing (WGS) found that *T. arvense* was highly homologous with *A. thaliana*. Based on this, the following comparative genomics and other gene-level mining centers on whole-genome sequencing are widely used in the research on pennycress.

An interesting study is about the pennycress nectary, which is the first step to improving the nectar yield. Most *Brassicaceae* flowers have two pairs of non-equivalent nectaries; however, *T. arvense* flowers develop four equivalent nectaries. Between immature and mature nectaries of pennycress, over 3,000 genes were identified to be expressed differentially in gene ontology and metabolic pathway analyses (Thomas et al., 2017). The sugar yield of pennycress is much lower than camellia and canola, but it has a higher pollinator visit time (pvt) value because of its additional feed value to pollinating insects (Eberle et al., 2015). In addition, comparing the nectary transcriptome of pennycress and *Arabidopsis*, it is suggested that the mechanism of nectary maturation and nectary secretion of the two plants is highly conservative. Sucrose phosphate synthetase, which is necessary to produce nectar in *A. thaliana*, was not highly expressed in pennycress. Nevertheless, an *A. thaliana SUCROSE SYNTHASE1* (*SUS1*, Ta03482) homologous gene is profoundly expressed in mature pennycress nectaries. Therefore, Thomas et al. (2017) speculated that Ta03482 may replace the function of sucrose phosphate synthase in full-fledged pennycress nectaries.

Based on the results of comparative genomics, WGS, and co-segregation analysis, Dorn identified four natural alleles of FLC in pennycress that imply a spring annual growth habit (Dorn et al., 2018). The result suggested that mutation in FLC is the key factor leading to the loss of vernalization requirement in spring annual lines. Geng et al. (2021) *de novo* sequenced and assembled the genome of *T. arvense* from Kunming (southwest of China) at the chromosome level and detected genes related to DNA repair, ubiquitin system, and high-altitude adaptation. Notably, the *FLC* gene undergoes strong natural selection under a high altitude environment, and the functional loss of FLC protein caused by a single base mutation may cause premature flowering (Geng et al., 2021). The transcriptome analysis of *T. arvense* showed that the uplift of the Qinghai Tibet Plateau (QTP) had no significant impact on the population dynamics of *T. arvense*, which may be due to the rapid propagation of *T. arvense* seeds (An et al., 2015). However, natural selection in extreme habitats will prompt epigenetic differentiation among populations. *T. arvense* growing in extreme natural environmental conditions like low temperature, low oxygen, and high UV irradiation in the Tibetan Plateau of China has experienced strong natural selection; thus, the related genes have been highly differentiated (Guan et al., 2020). Hu et al. (2021) assembled a high-quality *T. arvense* genome and revealed the mechanism of plant response to extreme environments mediated by reverse transcriptional replication.

The RNA of 22 pennycress accessions derived from embryos at two developmental stages was analyzed using RNA-seq (García Navarrete et al., 2022). The data from this analysis support that the accession Ames 32872, originally from Armenia, is highly divergent from the other accessions, while the accessions originating from Canada and the United States cluster together.

The existence of highly similarity between the *Arabidopsis* and pennycress genomes has been determined by comparative genomics as well as the existence of a largely one-to-one correspondence between them. Using a shotgun mutagenesis approach, a *Ta*-*max3*-*like* dwarf mutant and *Ta*-*kcs5/cer60*-*like* wax mutant deficient in the long-chain fatty acid biosynthesis were also identified (Chopra et al., 2018). Methane has been recognized as the second greenhouse gas after carbon dioxide, which contributes to global warming (Keppler et al., 2006). In addition to anaerobic sources, plants can also emit CH_4_ under aerobic conditions, which may harm the global methane budget. CH_4_ emission mainly comes from plant vegetative parts and rarely from reproductive parts (Qaderi and Reid, 2014). In addition, stressed plants emit more methane than non-stressed plants, and the emission rate varies with plant species (Bruhn et al., 2012; Martel and Qaderi, 2019).

As an oilseed crop in the fallow period, pennycress appears in various crop rotation systems frequently, which will bring some economic benefits. A pennycress–soybean double-crop system can not only increase the total seed yield but also reduce the weed pressure in the cropping system (Johnson et al., 2015)—that is to say, pennycress stands established in autumn and will grow rapidly in spring to control early weed species (such as common lamb quarters and giant ragweed) and late weed species (such as tall water hemp) (Johnson et al., 2015). In the double-cropping system of *T. arvense* and camellia (*Camelina sativa* L. Crantz) in Germany, the flower pollination of *T. arvense* mainly depends on wind pollination, the contribution of self-pollination is only half of that of wind pollination, and the role of insect pollination is less (Groeneveld and Klein, 2014). Simultaneously, *T. arvense* provides abundant forage resources for different insect groups due to the fact that other plants rarely bloom in the same season.

To be honest, the growth of pennycress will also bring some other ecological changes. It is well known that there is a symbiotic relationship between *Thlaspi* and AMF fungi. Dean et al. (2017) characterized the fungi community, except AMF for pennycress, for the first time and found that the diversity and richness of the fungi community of soybean licking with pennycress as the covering crop were higher. In the corn–soybean cropping rotation system, pennycress is the substitute host of soybean cyst nematode (SCN; *Heterodera glycines*), whereas even at high incipient population density, SCN has no significant influence on the biomass of soybean and pennycress (Warwick et al., 2002; Hoerning et al., 2022).

## 7 Research on the abiotic stress of pennycress

Several studies have found that *T. arvense* responds to various abiotic stresses. Sharma et al. (2006) used microarray technology to discover that the gene response pattern of *T. arvense* under chilling stress remarkably resembles the model plant *Arabidopsis* and exhibits greater chilling tolerance compared to *A. thaliana* and *B. napus*. According to gene functional annotations and algorithms, 595 genes in *T. arvense* showed upregulation or downregulation under cold stress. The most striking observation is that the S-adenosylmethionine (AdoMet) gene is upregulated in the pathway controlling sulfate assimilation, which shows that increasing AdoMet may be an effective strategy for *T. arvense* to cope with cold stress. The CBF/DREB family is widely known to be involved in regulating cold stress-related transcription factors in various species. According to research, pennycress showed superior cold tolerance than other *Brassiceae* plants after 3 weeks of cold acclimation (Zhou et al., 2007). Moreover, the overexpression of the *TaCBF* gene identified in *T. arvense* enhanced the cold resistance of *Arabidopsis* (Zhou et al., 2007).

The influence of salinity (NaCl) stress on the epigenetic variation of DNA methylation in field *T. arvense* was investigated by using methylation-sensitive amplification polymorphism (MSAP) markers (Geng et al., 2020). Salt stress stimulation increased the apparent genetic diversity of the *T. arvense* population, and this stimulation could be partially transmitted to the offspring under a non-stress environment in the manner of stress memory. In addition, parallel changes in functional traits were observed, that is, the phenotypic variation of plants under salt stress was critically much higher than that of the control group.

In agreement with the findings of previous studies on *Arabidopsis*, it was observed upon extensive characterization of the *TRANSPARENT TESTA* 2 (*tt2*) mutant line that *tt2* can be susceptible to a number of abiotic stresses including osmotic stress, drought, and freezing (Chen et al., 2012; Ott et al., 2021).

The flow cytometric analyses of the leaves of two *Thlaspi* species suggested that *T. arvense* is a non-accumulator plant, while alpine pennycress (*Thlaspi caerulescens*) is a hyper-accumulator (Monteiro et al., 2010). No significant absorption or accumulation differences were detected in the determination of cadmium (Cd) and zinc (Zn) distribution in *T. arvense* leaves by laser ablation-inductively coupled plasma mass spectrometry (LA-ICP-MS) (Galiová et al., 2019). Since LA-ICP-MS is not sensitive to non-accumulator plants, it has been proved that pennycress is not super-accumulated to Cd and Zn. Further research found that Cd stress led to a decrease in water content, osmotic pressure, chlorophyll a and b content, and photosystem II efficiency in pennycress leaves and also led to nutrient imbalance in *T. arvense* roots (Monteiro and Soares, 2021).

Pennycress is sensitive to copper and is a suitable representative in understanding how common dicotyledons deal with middle-level metal pollution in urban ecosystems (Manceau et al., 2013). The analysis combined with synchronous X-ray fluorescence and absorption techniques showed that the concentration of copper in the roots of pennycress was 50–100 mg/kg, which exceeded the cell demand, accumulated in the cell wall of cortical and stellar root cells, and combined with nitrogen and oxygen donors in histidine residues (Manceau et al., 2013). Moreover, *T. arvense* is a super-rich selenium plant. Selenium polysaccharides with antioxidant and anti-tumor effects can be extracted from the leaves by efficient subcritical water extraction (SWE) (Xiang et al., 2022).

## 8 Extraction and content improvement strategy of pennycress seed oil and fatty acid

Pennycress seeds are composed of oil, protein, glucosinolates, tannins, fiber, and many other secondary metabolites, of which glucosinolates (about 100 µmol/g of seeds) were all in the form of sinigrin virtually (Warwick et al., 2002; Chopra et al., 2020a).

The high residue on the soil surface will also lead to the reduction of the establishment of grass–clover forests, which was observed in South Dakota in 2013 and 2014 (Eberle et al., 2015). Apart from genetic factors, the oleaginousness of field pennycress is also limited with environmental factors like soil temperatures and total rainfall (Cubins et al., 2019). Several research pointed that pennycress yield is closely related to soil temperature and humidity (Johnson et al., 2015; Zanetti et al., 2019). The oil accumulation of pennycress seeds is positively correlated with the vegetation period. Planting seedlings in the northern corn belt from late August to September will maximize the output and oil content (Dose et al., 2017). The higher accumulative rainfall, accumulative photohydrothermal period, and higher soil temperatures during sowing provide favorable environmental conditions for long-term growth at the cost of reducing the crude protein of the seeds so as to maximize the oil content (Dose et al., 2017). It was discovered that the seed thickness, 1,000-grain weight, oil content, peroxide value, acid value, and monounsaturated fatty acid content of *T. arvense* seeds were related to longitude and latitude, annual average rainfall, annual average temperature, and other geographical environments (Liu et al., 2022). Seed germination is also an important indicator for evaluating seed oil content. The total germination rate of seeds from different regions varies greatly, which has a significant impact on seed oil content and variety selection (Edo-Tena et al., 2020). In addition, Cubins et al. (2022) demonstrated that delaying the seed harvest time would cause only 26% of the loss, ensuring the maximum yield of pennycress seeds and the minimum loss of oil content at harvest time.

Same as the oil content, the erucic acid content was significantly different in different positions (*p* < 0.05), but it did not increase significantly through position interaction. Gesch et al. (2016) believed that there were variations in fatty acids in different locations, and they found that there were crucial differences in erucic acid in a single population in two regions. On the contrary, Claver et al. (2017) put forward the opposite view since there was no visible distinction in the oil characteristics of *T. arvense* seeds planted in the growth chamber and in field conditions.

As a newly developing oilseed crop, pennycress has irreplaceable advantages over other oilseed crops. Under the same conditions, the growth cycle of pennycress is shorter than that of other oil crops, which is conducive to establishing a double-cropping system to replace winter fallow (Zanetti et al., 2019). However, differently from the conclusion of Dose et al. (2017); Zanetti et al. (2019) found that there was a strong association between the seed yield and the seed oil content of pennycress.

It is undeniable that the breeding of pennycress has just started and that it still has many characteristics that need to be improved. At present, *T. arvense* can be domesticated and engineered using a variety of molecular biological methods. The characteristics of weeds, such as seed dormancy and long survival time, are currently the target of domestication *via* CRISPR-Cas9 gene editing and mutation breeding techniques (Sedbrook et al., 2014). Screening and tendentious cultivation of these characters in pennycress will help to increase the net income and realize oilseed covering crops (Ott et al., 2019). The *TaAOP2* gene catalyzes the last step of the pennycress glucosinolate pathway. The mutation of this gene leads to the reduction of the metabolic flux of the pathway, but the specific mechanism is still unclear (Chopra et al., 2020b). The simultaneous mutation of both *TaROD1* (*REDUCED OLEATE DESATURATION1*) and *TaFAE1* (*FATTY ACID ELONGATION1*) genes can make the oleic acid content produced by pennycress much higher than that of the single mutants (Chopra et al., 2020a). The lipid profile of *T. arvense* with a double mutation of the *fad1* (*FATTY ACID DESATURASE2*) *rod1* gene is similar to those of canola seed TAGs, while the growth of plants with *fad2* single mutation and *fad2 fae1* double mutation was slow, and the overall height and seed yield decreased (Jarvis et al., 2021). More experiments are needed to explore the response of this genotype plant under various biological and abiotic stresses. The introduction of the acyltransferase (*LPAT* and *diacylglycerol acyltransferase*) gene and thioesterase (*FatB*) gene of its non-related species *Cuphea* into pennycress promoted the accumulation of a large amount of medium-chain fatty acids (MCFAs) in seeds without affecting the seed vitality (Esfahanian et al., 2021). According to the metabolomics and transcriptomics analysis of the 22 species of pennycress, improving the oil content of pennycress seeds can be achieved through four effective strategies: chlorophyll carbon allocation, gene modification related to lipid synthesis, enhancement of embryonic photosynthetic efficiency, and strict control of nitrogen utilization (Arias et al., 2023).

## 9 Conclusion

Pennycress is a promising new oilseed crop. There are many studies on the extraction technology of pennycress fuel. As an oil crop, it still has many characteristics that need further domestication and screening to increase its adaptability to the ecosystem. Recently, more scholars have contributed many excellent achievements to the research on pennycress species. Although several scholars have sequenced and assembled the genome of *T. arvense*, the function and various mechanisms of the *T. arvense* gene have not been fully explored. In particular, there is still a large gap in the research on pennycress in response to abiotic stress and the expression and regulation of related genes. Moreover, issues such as the standardized and unified planting standards for pennycress and the removal of sinigrin as proposed by Cubins et al. (2019) have not yet been resolved. There are many aspects to be solved in future research, such as the assembly and supplementation of genomes, combined with the analysis of the mechanism of regulating the fatty acid synthesis pathway in multi-omics, the cultivation of “zero erucic acid” pennycress seeds with healthy diet, or breeding the “high erucic acid” pennycress seeds for biofuels.

## Author contributions

JM: Writing – original draft, Writing – review & editing. HW: Writing – review & editing. YZ: Writing – review & editing.
